# The impact of e-cigarette exposure on metabolic function: a narrative review

**DOI:** 10.3389/fpubh.2026.1930924

**Published:** 2026-09-30

**Authors:** Insiya Sajjad Hussain Saifudeen, Sama Thiab, Abrar Alhermi, Stephen L. Atkin, Alexandra E. Butler

**Affiliations:** 1 School of Medicine, Royal College of Surgeons in Ireland, Busaiteen, Bahrain; 2 Research Department, Royal College of Surgeons in Ireland, Busaiteen, Bahrain

**Keywords:** cardiovascular effects, diabetes, electronic cigarette, electronic nicotine delivery systems, insulin resistance, metabolic syndrome, nicotine, obesity

## Abstract

Electronic cigarettes (e-cigarettes) have rapidly emerged as a popular alternative to conventional tobacco smoking, contributing to a substantial rise in their global use over the past decade. Although marketed as a safer, combustion-free method of nicotine delivery, increasing evidence suggests that e-cigarette exposure may adversely affect metabolic health. This literature review evaluates current evidence regarding the impact of e-cigarette use on the metabolic system. Relevant studies published until 2026 were identified through searches of databases including PubMed, Embase, CINAHL, and reports from the World Health Organization. This narrative review incorporates findings from both human and animal studies examining the metabolic consequences of e-cigarette exposure. Current evidence suggests that nicotine and other aerosol constituents may contribute to metabolic dysregulation through oxidative stress, inflammation, mitochondrial dysfunction, and altered cellular signaling pathways. Several studies have demonstrated associations between e-cigarette use and obesity-related outcomes, including increased body mass index and adipose tissue dysfunction. Evidence has also linked e-cigarette exposure to insulin resistance, impaired glucose metabolism, elevated HbA1c levels, reduced antioxidant activity, and increased expression of glycogen synthase kinase-3 beta (GSK3β), a pathway implicated in the development of prediabetes. Additionally, broader components of metabolic syndrome, including hypertension, dyslipidemia, endothelial dysfunction, and cardiovascular risk factors, have been explored in the literature. While the findings collectively suggest a potential relationship between e-cigarette use and adverse metabolic outcomes, the current body of evidence remains limited by the predominance of cross-sectional and short-term studies. Further longitudinal, clinical, and mechanistic research is necessary to better establish causality and clarify the long-term metabolic effects of e-cigarette use.

## 1 Introduction

Electronic cigarettes (e-cigarettes) are compact, battery-powered vaporizers designed to deliver nicotine vapor. They heat a solution that typically includes nicotine, propylene glycol (PG) or glycerol, and flavorings, producing an aerosol that users inhale, as shown in Figure 1 (1). Unlike traditional tobacco products, e-cigarettes vaporize nicotine without combustion. Devices vary in features such as disposability, rechargeability, control over puff delivery and temperature, and e-liquid composition and nicotine concentration (2). These devices are commonly referred to as vapes (2). E-cigarettes have seen explosive growth in popularity, with an estimated 68 million users globally in 2020 (3). The global market value increased from $7.8 billion in 2015 to $22.3 billion in 2022 (4). Their appeal is multifaceted, driven by perceived safety, flavors, and targeted marketing (5). Originally introduced as alternatives to traditional smoking, e-cigarettes are promoted as less harmful ways to consume nicotine. However, rapid adoption, particularly among young people, has raised concerns about potential health risks (5, 6). When vaporized, e-cigarette liquids can generate chemical byproducts that may adversely affect physiological processes, with emerging concerns regarding metabolic health (6–10).

**Figure 1:**
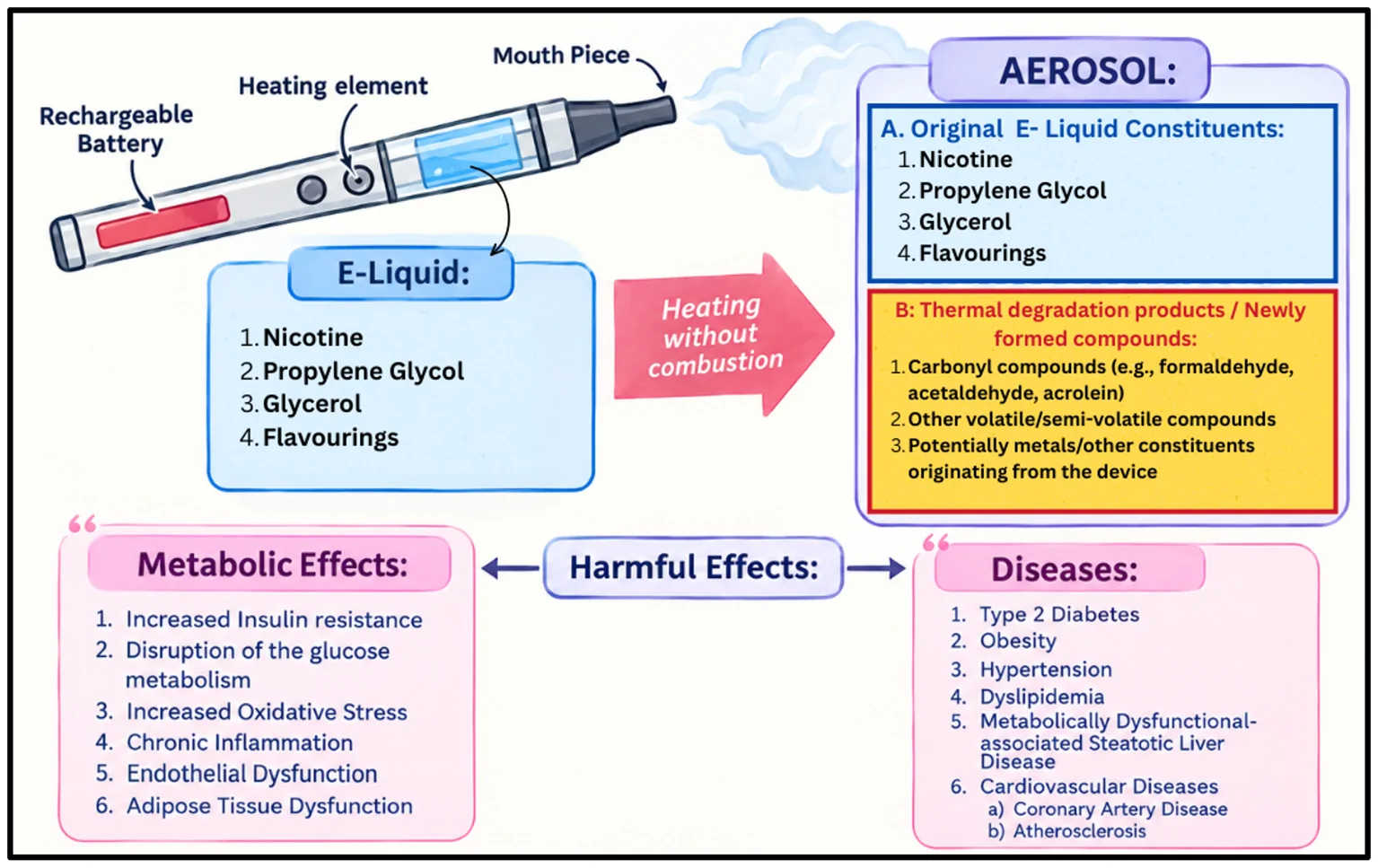
Overview of the harmful metabolic effects of e-cigarettes (1, 7, 9).

Nicotine is a naturally occurring but highly addictive alkaloid of the Solanaceae family, primarily concentrated in tobacco plants and incorporated into products such as cigarettes, cigars, smokeless tobacco, hookah, e-cigarettes, and heated tobacco devices (11). E-cigarettes are marketed in a wide range of nicotine concentrations, from 0 mg/mL up to >60 mg/ml in certain formulations (12). Patterns of exposure are typically quantified either by liquid concentration or by consumption units (e.g., cartridges, pods). However, nicotine delivery efficiency varies substantially across device types, influencing both dependence potential and physiological outcomes (11, 12).

The metabolic system maintains energy homeostasis and overall health through biochemical pathways regulating nutrient conversion, lipid storage, and glucose balance (8). Disruptions in processes such as glycolysis and insulin signaling are closely linked to obesity, type 2 diabetes (T2D), and cardiovascular disease (8, 9). Nicotine from traditional tobacco products has been shown to impair glucose metabolism, induce insulin resistance, and alter lipid profiles (9). However, the metabolic consequences of nicotine delivered via e-cigarettes remain unclear, particularly given differences in dose, device type and user behavior.

In addition to nicotine, e-cigarette liquids contain PG, glycerol, and flavorings, which can produce reactive oxygen species (ROS) and other byproducts when heated (7, 10). While these compounds have been associated with oxidative stress and inflammatory responses, their direct effects on metabolic pathways are still being elucidated. This knowledge gap highlights the need to examine both nicotine and other e-cigarette constituents in relation to metabolic health. This literature review synthesizes current research on the effects of e-cigarette use on the metabolic system, including obesity, insulin resistance, glucose regulation, lipid profiles and cardiovascular outcomes. By evaluating existing evidence, the review identifies areas of emerging evidence alongside important gaps in knowledge, providing guidance for future research and public health considerations.

## 2 Methodology

This narrative review was conducted in accordance with established guidelines for structured literature synthesis. A comprehensive search was performed across four electronic databases: PubMed, Embase, CINAHL, and the World Health Organization (WHO) Global Index Medicus. The search covered all available literature from database inception to March 2026.

A combination of Medical Subject Headings (MeSH) terms and free-text keywords was used to capture relevant studies. Search terms included: “e-cigarette,” “electronic cigarette,” “electronic nicotine delivery systems,” “ENDS,” “vaping,” “nicotine,” and “non-nicotine,” combined with terms related to metabolic and cardiovascular outcomes, including “metabolic syndrome,” “obesity,” “body mass index,” “diabetes,” “prediabetes,” “HbA1c,” “insulin resistance,” “glucose metabolism,” “lipid profile,” “dyslipidemia,” and “cardiovascular biomarkers.”

### 2.1 Eligibility criteria

The eligibility criteria for study inclusion and exclusion are presented in Table 1.

**Table 1:** Eligibility criteria for study inclusion and exclusion.

Inclusion criteria	Exclusion criteria
1. Original research evaluating the effects of e-cigarette exposure or use on metabolic, endocrine, or cardiovascular outcomes.	1. Studies that did not evaluate e-cigarette exposure or relevant e-cigarette aerosol constituents/mechanistic pathways linked to metabolic outcomes.
2. Human studies or experimental animal models.	2. Studies that did not report metabolic, endocrine, or cardiovascular outcomes.
3. Exposure to nicotine-containing and/or nicotine-free e-cigarette formulations.	3. Non-original articles, including editorials, commentaries, letters, opinion pieces, or conference abstracts without primary data.
4. Studies reporting outcomes related to metabolic syndrome, obesity, insulin resistance, glucose metabolism, diabetes, lipid metabolism, inflammatory markers, or cardiovascular risk biomarkers.	4. Duplicate publications with overlapping datasets and no additional analyzable data.
5. Studies investigating e-cigarette aerosol constituents or shared mechanistic pathways relevant to metabolic dysfunction (e.g., nicotine and other toxicants), where applicable for mechanistic interpretation.	5. Studies with unavailable full text or insufficient methodological detail.
6. Studies published in the English language.	

## 3 Study selection

All retrieved records were imported into a reference management system and screened for relevance. Titles and abstracts were initially assessed for eligibility, followed by full-text review of potentially relevant articles.

Study selection was performed independently by one reviewer and verified by a second reviewer to ensure consistency and reduce selection bias. Any disagreements were resolved through discussion and consensus. Given the narrative nature of this review and its broad scope, study selection focused on identifying relevant evidence across the predefined metabolic, endocrine, and cardiovascular domains rather than following a formal systematic-review screening protocol.

## 4 Data extraction and synthesis

Data were extracted using a standardized approach, including study design, population characteristics, type and duration of exposure, metabolic outcomes assessed, and key findings.

Given the broader scope of the present evidence synthesis and the heterogeneity in study designs, exposure assessment methods, and outcome measures, a narrative review approach was selected for this evidence synthesis instead of a scoping or systematic review, which would be more suitable for addressing a focused research question on the potential adverse impact of electronic cigarette use on metabolism. Therefore, a meta-analysis was also not performed, and the findings from the identified studies were synthesized narratively and structured according to key metabolic domains, including obesity, insulin resistance and glucose metabolism, lipid metabolism, metabolic syndrome, and cardiovascular disease risk, as presented in the following sections.

### 4.1 E-cigarette composition and mechanisms of action

#### 4.1.1 Nicotine and metabolic dysregulation

Nicotine appears to play an important role in the metabolic effects associated with e-cigarette exposure, although its contribution may vary depending on the exposure model and the presence of other e-cigarette constituents. Evidence from animal and human studies has identified several potential pathways through which nicotine may influence metabolic health (13–19).

##### 4.1.1.1 Mechanistic insights from animal studies

Preclinical research has elucidated multiple pathways suggesting potential links between nicotine exposure and metabolic dysregulation. Genetic variations in nicotinic acetylcholine receptor (nAChR) genes, especially the α3/α5/β4 cluster on chromosome 15, modulate susceptibility to nicotine dependence and T2D, in part via altered regulation of blood glucose through medial habenula (MHb) nAChR activity (13). Chronic nicotine stimulation in animal models induces sustained hyperglycemia resembling T2D (13). Nicotine also inhibits hypothalamic AMP-activated protein kinase (AMPK), decreasing food intake while increasing energy expenditure (13). At the endocrine level, nicotine elevates catecholamines and cortisol, antagonizing insulin signaling and promoting insulin resistance (13).

Animal studies have further shown nicotine's role in lipid metabolism. It promotes lipolysis and fatty acid transport to the liver and muscle, enhancing VLDL secretion and intramyocellular lipid accumulation, thereby impairing insulin sensitivity (13, 14). *In vitro*, nicotine stimulates mTOR/p70S6K signaling, leading to IRS-1 serine phosphorylation and reduced glucose uptake (13). In rodents, both nicotine-containing and nicotine-free e-liquids disrupt glucose regulation, with e-cigarette aerosols producing more pronounced hepatic stress than pure nicotine exposure (14, 15). Evidence also links chronic e-cigarette exposure to hepatic oxidative stress, lipid accumulation, necrosis, and circadian rhythm disruption—effects observed in metabolic dysfunction-associated steatotic liver disease/metabolic dysfunction-associated steatohepatitis models and potentially mediated by mitochondrial dysfunction and DNA damage (15).

##### 4.1.1.2 Evidence from human studies

In humans, acute nicotine exposure stimulates catecholamine release and lipolysis, as evidenced by increases in circulating adrenaline, glycerol, and free fatty acids following intravenous nicotine administration in fasting healthy men (16). Observational evidence suggests that smokers generally weigh 4–5 kg less than non-smokers, likely due to nicotine's sympathomimetic effects, which enhance lipolysis, thermogenesis, and energy expenditure (17). Nicotine may also influence appetite-regulating pathways and circulating leptin levels, although evidence regarding its effects on leptin is mixed (18). However, repeated nicotine exposure may lead to tolerance to some of its metabolic effects, potentially modifying its long-term effects on energy expenditure (19).

#### 4.1.2 Non-nicotine components of e-liquids and their metabolic effects

Non-nicotine constituents include the liquid solution consisting of 1,2-propanediol (PG), vegetable glycerin (VG), and flavorings. The battery-powered heating element produces an aerosol out of these components that the user can inhale (20, 21).

The primary and prevalent constituents in e-liquids are PG and glycerol. These compounds serve as humectants, critical in preventing the e-liquid from dryness (22). A study investigated the effects of e-cigarette aerosols containing glycerol as the vaping liquid on both adult and young male and female mice. Results have shown that inhaled glycerol from e-cigarette use can enter the bloodstream and lead to transient elevation of circulating glycerol levels, particularly in females. It was observed that female mice exhibited higher levels of hepatic triglycerides and phosphatidylcholine without any noticeable alterations in the liver's health (23). A study on e-cigarette constituents' metabolism in airway epithelial cells *in vitro* has demonstrated that short-term exposure to PG and VG reduced GLUT-mediated glucose transport and ATP production in airway cells. The observed phenomenon can be attributed to alterations in cell volume and membrane fluidity induced by osmotic influences and the direct interaction between PG/VG and lipids present in the cell membrane (24).

The addition of flavor to e-cigarettes has been determined as the main factor driving the use of e-cigarettes among 81% of young users (25). It intends to enhance the product's attractiveness, pleasure, and overall user fulfillment (25). A study examining the cytotoxic effects of flavoring compounds in an *in vitro* setting revealed that cinnamaldehyde (CAD) exhibited significant inhibition of metabolic activity. Similarly, anethole and anisaldehyde were observed to suppress cellular metabolic activity substantially (26). Furthermore, exposure to small amounts of formaldehyde, a flavoring component, in animals has also been demonstrated to cause a reduction in blood pressure. This decrease is believed to result from formaldehyde directly affecting the smooth muscle cells (26).

In a study examining various flavoring chemicals, CAD exhibited the highest level of toxicity, while other flavors, such as O-vanillin and pentanedione, also demonstrated notable cytotoxicity. The study revealed that combining several flavors or using them in proportions increases the toxicity levels. Moreover, the tested flavors generated significant levels of ROS in a cell-free environment, indicating a potential mechanism for their cytotoxic effects (22).

Human research also indicates that flavors influence vaping patterns and nicotine exposure. While fruit and menthol flavors were associated with greater puffing compared to tobacco flavor, nicotine—not flavor—was the main factor reducing withdrawal symptoms (27).

Additionally, pod-style e-cigarettes using nicotine salts deliver higher nicotine levels with less irritation than free-base nicotine. At 40 mg/ml, nicotine salts can achieve blood nicotine concentrations comparable to cigarette smoking, raising concerns about addiction potential and regulation (28).

### 4.2 E-cigarettes and obesity

While an inverse relationship between tobacco smoking and body mass index (BMI) has already been established (29), the link between e-cigarette use and weight status remains poorly understood. Nicotine has long been hypothesized to have an appetite suppressing effect (15), given its rapid delivery to the brain and subsequent release of neurotransmitters associated with appetite and pleasure (e.g., serotonin, dopamine, norepinephrine) (30). It is also a known inhibitor of lipogenesis, such that with increased lipolysis, upregulation of thermogenesis in fatty tissue results and may be an underlying mechanism behind which nicotine exhibits its weight reducing effects (30). This concept may apply at the level of both tobacco and e-cigarette use; however, e-cigarette use and smoking is each comprised of different nicotine concentrations, delivery methods, and other chemicals (i.e., tobacco vs. different additive flavors, respectively) that could lead to weight loss, but potentially through separate mechanisms (31, 32). In a cross-sectional study of 965 patients (average age: 50 years; average BMI: 30.8 kg/m^2^), e-cigarette use showed robust association with a lower BMI (*p* = 0.021) in survey respondents controlled for their age, gender, education, and ethnicity (30). Specifically, this negative association displayed a *U*-shaped relationship in upper and lower BMI quantiles, such that e-cigarette use was more consistently associated with BMI classes in both extremes (30). Reports of those who use e-cigarettes have been noted in individuals with intention to lose weight, whereby several studies have shown an increased usage of e-cigarettes in those who have weight concerns (30–32). This finding may confound an apparent link between e-cigarette users and a low BMI, whereby a change in their lifestyle (e.g., unhealthy calorie restriction, spurious thinness, laxative intake) may be the main driver behind their weight loss and/or weight status, rather than it solely being due to e-cigarette usage. Perhaps certain flavors and aromas augment satiety and subsequently promote a reduction in food intake, highlighting the need for further research investigating this phenomenon.

Evidence for a strong association between e-cigarette use and weight gain remains unclear. Indirect evidence of a lack of association between these two variables was shown in an *in vitro* study of a 3T3-L1 cell line, whereby e-cigarettes were not found to increase induction of differentiation of preadipocytes to beige or brown adipocytes, relative to conventional smoking (33). This biologically plausible mechanism suggests that a multitude of factors, besides nicotine, could be underlying the metabolic effects of e-cigarettes (33). Ambiguity of data on e-cigarettes (i.e., content, type, nicotine concentration, flavor, dual usage) further complicates the lack of information regarding a clear effect, or lack thereof, on weight status.

Despite such hypotheses behind a possible link between e-cigarettes and a reduced BMI or weight status, we have identified an overwhelmingly broader evidence base for an association between obesity and e-cigarettes from recently conducted studies (34–38). In a cross-sectional study of 452 young adults (average age: 21.3 years ± 2.6), response analysis of a survey on health-risk behavior showed an association between belonging to the cigarette/electro nic tobacco use group and two weight indicators: deviation from demographic cohort's ethnic and gender BMI class norm and obesity (34). Specifically, more obese participants belonged to the cigarette/electronic tobacco use group (participants with low alcohol endorsement) than the “high substance use” group (participants with high alcohol endorsement, along with tobacco and electronic tobacco use), and the same was observed when looking at deviation from the group BMI norm. However, the directionality of that relationship is yet to be determined.

In an annually conducted cross-sectional study, Mason and colleagues examined trends of e-cigarette use among adolescents according to weight status and sex (35). Specifically, they determined an association of both female sex and increased weight status (overweight and obese) with e-cigarette use. This observation came in contrast with declining e-cigarette use trends among all males and specifically females of a healthy weight status, highlighting the importance of targeting particular demographics and BMI groups for preventative practice (35).

Less robust, yet still supportive, evidence comes from a rodent model study that also showed similar sex-specific findings pertaining to administration of nicotine and e-cigarette flavorings in diet-induced obese mice (36). Results showed more frequent administration of e-cigarette liquid, containing 6 mg/mL nicotine, menthol, and green apple chemical flavorants, in diet-induced obese female mice compared to their male counterparts. Similarly, diet-induced obese female mice exhibited greater self-administration of nicotine with menthol flavoring compared to female mice given unflavored nicotine of the same dose. These findings highlight the potential influence menthol-flavored nicotine e-liquids could have on initiation of smoking behavior in obese female cohorts (36).

Analysis of data from the National Health and Nutrition Examination Survey (NHANES) cycles across 4 years showed evidence supporting the association of passive exposure to both e-cigarette aerosol and cigarette smoke with overweight and obese BMI groups in 968 individuals (ages 3–19) (37). Second-hand exposure to e-cigarette aerosol and smoke was evaluated using self-reported measures, and serum cotinine levels were also assessed as an objective measure. Greater odds of being obese and overweight were seen in those passively exposed to both e-cigarette aerosol and cigarette smoke (adjusted odds ratio = 3.9, 95% CI: 1.4–11.0; *p* = 0.009), further suggesting an association between obese weight status and e-cigarette exposure (37). Serum cotinine levels were also associated with obesity risk in a dose-dependent fashion, with participants in the fourth serum cotinine quartile showing significantly greater adjusted odds ratio than those in the first quartile (adjusted odds ratio = 3.8; 95% CI: 2.1–6.8; *p* < 0.001). Similarly, Zhao and colleagues observed a significant association between e-cigarette use and obesity in their cross-sectional study (odds ratio = 1.6, 95% CI 1.3–2.1; *p* = 0.0007), further highlighting the evidence for the relationship between these two factors (38). Because of the cross-sectional nature of these studies, directionality of the relationship between e-cigarette usage and obesity is still poorly understood and makes the identification of underlying mechanisms behind this association of utmost importance.

Stress and depressive symptoms, especially those experienced in young adulthood and adolescence, that are attributed to obesity and an unhealthy lifestyle can predispose to e-cigarette use (e.g., for social acceptance); however, a confounding effect of e-cigarette use on appetite might instead be a major factor (i.e., affecting impulsivity/neurological reward pathways), drawing further necessity for future research aimed at understanding this relationship.

Table 2 summarizes key identified studies that explored the association of e-cigarette use and/or exposure with obesity, BMI status, weight loss, and other obesity-related variables.

**Table 2:** Summary of evidence table from studies reviewed pertaining to the association of e-cigarette use or exposure with obesity, BMI status, weight loss, and other related variables, including main exposure, identified outcomes, and key findings from each reviewed study.

Study	Study design	Population	Main exposure	Outcome	Key findings
Zagoriti et al. (33)	*In-vitro* study	3T3-L1 cell line	E-cigarette exposure	Preadipocyte differentiation to beige or brown adipocytes	E-cigarette exposure did **not** increase differentiation of preadipocytes to beige or brown adipocytes, compared to conventional smoking exposure
Tetteh-Quarshie et al. (36)	Rodent experimental study	Diet induced obese mice (male and female)	Administration of nicotine and e-cigarette flavorings (menthol, green apple flavorant)	Frequency of e-cigarette liquid self-administration and type of administration (nicotine with flavorants; nicotine alone)	1. Diet-induced female mice showed increased self-administration of e-cigarette liquid containing menthol, nicotine, and green apple flavorant compared to their male counterparts. 2. Greater self-administration of e-cigarette liquid containing nicotine with menthol in diet induced obese female mice than female mice in the unflavored nicotine group
Alqahtani et al. (30)	Cross-sectional (survey response)	965 adults (average age = 50; average BMI = 30.8 kg/m^2^)	E-cigarette use	BMI	E-cigarette use associated with BMI classes in both extremes, including association with lower BMI status (*p* = 0.021)
Morean et al. (31)	Cross-sectional (survey response)	529 adolescents (average age = 16.27 years; SD = 1.18)	E-cigarette use; vaping flavored e-liquid	Intention of weight loss and appetite control	Increased frequency of e-cigarette use and use of larger flavored e-liquid quantities was associated with intentions of weight loss and appetite suppression, respectively (*p* < 0.01)
Lanza et al. (34)	Cross-sectional (survey response)	452 young adults (average age = 21.3 years ± 2.6)	E-cigarette use	Obesity status and deviation from group's BMI norm	Association between e-cigarette use and belonging to the obese group and deviation from group BMI norm was found; although directionality was not determined
Mason et al. (35)	Annual cross-sectional study (survey response)	Adolescents (high school students)	E-cigarette use	Weight status and sex category	E-cigarette use association with overweight and obese status and female sex, whereas declining trend in females of a healthy weight and all males
Liu et al. (37)	NHANES database across four cycles; cross-sectional analysis	968 participants (age range: 3–19)	Passive exposure to e-cigarette aerosol and cigarette smoke (second-hand smoking)	Belonging to overweight and obese BMI groups	Passive exposure to e-cigarette aerosol and cigarette smoke (second-hand smoking) is associated with greater odds of being obese or overweight
Zhao et al. (38)	Cross-sectional (survey responses; two nationally representative Chinese datasets)	Adult participants (age > 18 years; 373,781 Chinese adults)	E-cigarette use	Obesity	Obesity was significantly associated with greater odds of e-cigarette use (OR = 1.6, 95% CI 1.3–2.1; *p* = 0.0007)

### 4.3 E-cigarettes and insulin resistance

Notwithstanding the impact of electronic nicotine delivery systems (ENDS) or e-cigarettes on cardiopulmonary health, their increased consumption among adolescents in the past decade has brought to light an added potential effect on metabolic profile. It has been established that the major component of e-cigarette fluid, nicotine, has been observed to directly elicit damage on isolated pancreatic islets and apoptosis of beta cells via nAchR, potentially leading to *in vivo* pancreatic dysfunction, which highlights the relationship between nicotine and insulin resistance (39). Cotinine, a primary biomarker for smoking exposure, was linked to an absolute increase in glycated hemoglobin A1c (HbA1c) among non-diabetic tobacco smokers with impaired fasting glucose (i.e., prediabetes) (40). Because cotinine is a major direct metabolite of nicotine, this observation suggests a broader association between nicotine and elevated HbA1c levels; however, causality cannot be determined due to the cross-sectional nature of survey-based studies. Interestingly, indirect evidence of this trend was observed by El Golli and colleagues in a rat model exposed to e-cigarette liquid without nicotine, which showed an induction of hyperglycemia, along with an upregulation of GSK3β in groups exposed to either e-liquid with nicotine or e-liquid without nicotine (15). This finding may potentially be linked to the sugars and aldehydes present within e-cigarette liquids, which typically contain a combination of glycerin and/or propylene glycol, that, when heated, give rise to the aforementioned aldehydes, namely acrolein, acetaldehyde, and aldehyde (41). These aldehydes have been linked to proinflammatory cytokine states, endothelial dysfunction, and dyslipidemia, thereby potentially explaining the results seen in the animal model study and suggesting a precipitating state that could, theoretically, lead to subsequent development of prediabetes and diabetes states (41).

A downregulation of cellular antioxidant levels, namely nuclear factor erythroid 2-related factor 2 (Nrf2), is also induced by nicotine, such that a resultant state of heightened oxidative stress may lead to insulin resistance via impaired insulin signaling and subnormal glucose uptake (42). Insulin resistance, an impaired biological response of target cells to normal insulin exposure, can be observed in diabetes and/or a preceding state of intermediate hyperglycemia, known as prediabetes. According to the American Diabetes Association (ADA), prediabetes can be categorically defined as either: 100–125 mg/dl fasting plasma glucose, 140–199 mg/dl plasma glucose 2 h after 75 g oral glucose tolerance test, or an HbA1c between 5.7% and 6.4% (43). Insulin resistance is a common underlying etiology in the development of prediabetes and diabetes, including their associated plethora of microvascular complications (namely diabetic retinopathy, nephropathy, and neuropathy); as such, the proposed association between e-cigarettes and insulin resistance can be drawn to both metabolic states. An overview of the potential pathogenic effects of e-cigarettes on insulin resistance is further illustrated in Figure 2.

**Figure 2:**
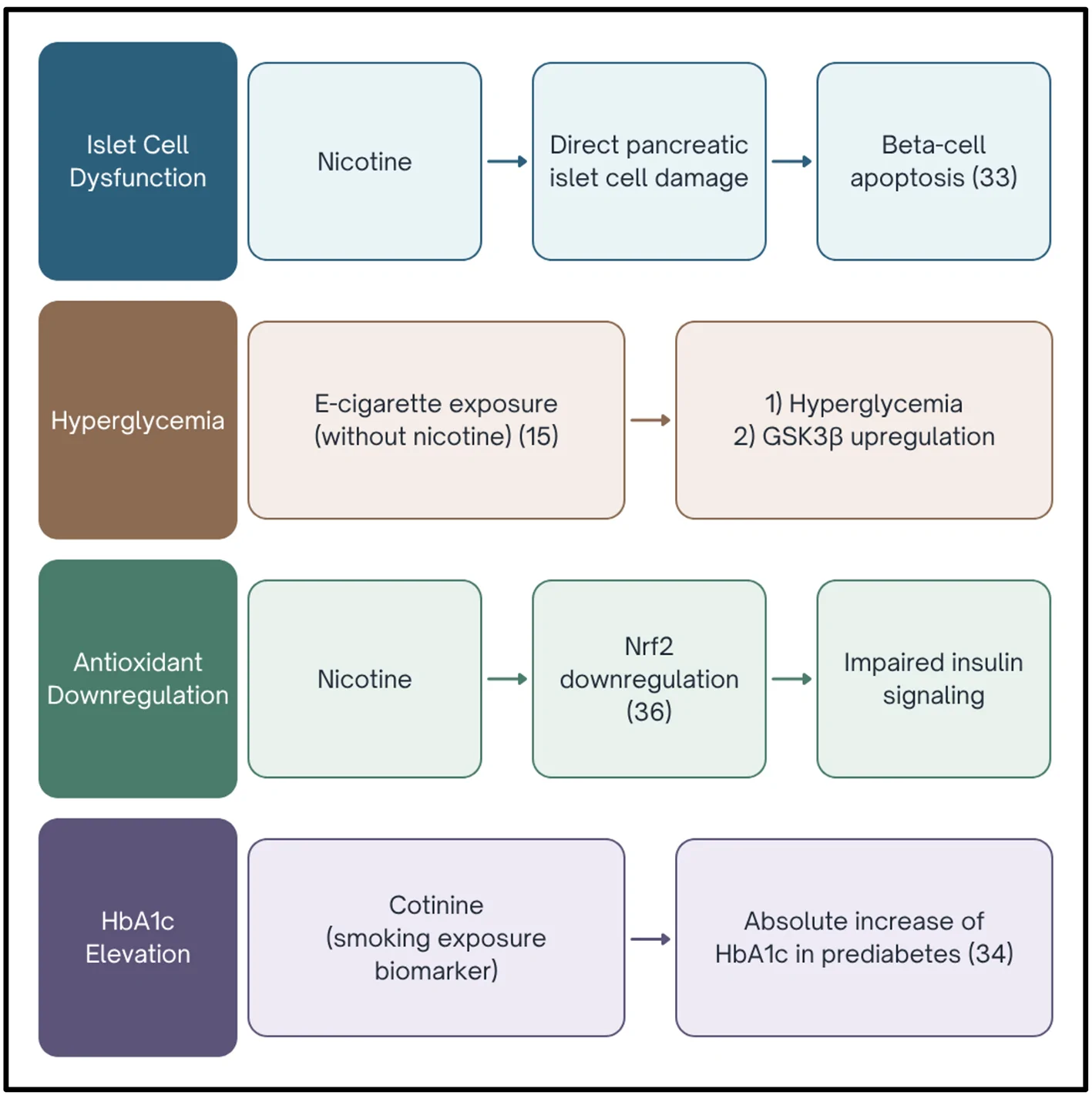
Overview of the potential associations of e-cigarettes with effects implicated in the pathogenesis of insulin resistance through different mechanisms and cellular pathways, namely Nrf2, GSK3b, HbA1c, and islet cell apoptosis (15, 39, 40, 42). Each displayed arrow represents an association rather than a causal relationship.

We identified supportive evidence from two main cross-sectional studies that observed an association between e-cigarette use and prediabetes in never-tobacco-smokers, using data from the Behavioral Risk Factor Surveillance System (BRFSS), a nationally representative database run between the Center for Disease Control and Prevention (CDC) and states in the USA (44, 45). Atuegwu and colleagues (44) found an increased odds ratio of prediabetes (self-reported) in those currently smoking e-cigarettes compared to former e-cigarette users (1.97, 1.25–3.10 95% CI vs. 1.07, 0.84–1.37 95% CI; respectively). Upon gender stratification, a persistently increased odds ratio was also observed in current e-cigarette users and former e-cigarette users (2.36, 1.26–4.40 95% CI vs. 1.22, 0.86–1.74 95% CI; respectively), when compared to never smokers. Similarly, Zhang and colleagues determined an increased odds of self-reported prediabetes among e-cigarette users in the very same representative sample of the US (BRFSS database) (45). Most notably, sole e-cigarette users (current) showed an increased odds ratio of prediabetes compared to dual users (users of combustible cigarettes and e-cigarettes) (1.54, 1.17–2.0 95% CI vs. 1.14, 0.97–1.34; respectively). Another study by Atuegwu and colleagues (46) reported on several new factors they found to be associated with an increased odds of sole e-cigarette usage, among which were obesity and a history of diabetes. However, both research groups adopted a cross-sectional study design with less robust evidence in self-reported measures of prediabetes, rather than laboratory tests, necessitating future research with prospective study designs to investigate a potential dose-response association between prediabetes risk and e-cigarette usage. Conversely, a nationally representative Scottish Health Survey found no clear association between current or former e-cigarette use and self-reported physician-diagnosed diabetes status (47). The greatest odds of diabetes, notably, were found in former tobacco smokers (odds ratio = 1.35, 95% CI = 1.14–1.60), compared to former e-cigarette smokers and current smokers. This finding may be attributed to a combination of prolonged tobacco smoking exposure and reverse causation, whereby patients would typically opt to quit following diagnosis (47).

To eliminate recall bias effect from self-reported measures in the BRFSS studies, another cross-sectional study utilizing laboratory confirmed measures of diabetes was conducted from the National Health and Nutrition Examination Survey (NHANES) (48). Conversely, this study showed no significant association of sole e-cigarette usage and dual smoking with diabetes and prediabetes among the general US population in the NHANES study (*p* > 0.05). Inconsistency of this finding with the previous two studies may be attributed to the self-reported vs. laboratory confirmed measures of prediabetes. However, the insulin resistance index, homeostatic model (HOMA-IR), was found to have 22% greater odds of being higher in former e-cigarette users than never e-cigarette users (adjusted odds ratio = 1.22, 95% CI: 1.00–1.84) (48). Another cross-sectional study examining the Korean National Health and Nutrition Examination Survey (KNHANES) also found an increased risk of prediabetes in dual smokers and sole tobacco smokers compared to non-smokers without second-hand smoking exposure (49). Interestingly, Kim and colleagues observed former dual smokers (e-cigarette and combustible cigarette smoking) to have a greater prediabetes risk than former sole combustible cigarette smokers (1.54, 95% CI: 1.04–2.31 vs. 1.04, 95% CI: 0.91–1.91) (49). When stratified according to female gender, no apparent association was found between smoking behavior and diabetes among participants who were women. Another study analyzing the KNHANES database found similar association between dual e-cigarette and tobacco use and likelihood of metabolic syndrome in 15,359 adults aged over 19 years (50). Greatest odds of metabolic syndrome likelihood was observed in dual smokers (odds ratio = 1.48, 95% CI 1.12–1.94), followed by cigarette-only users (odds ratio = 1.19, 95% CI 1.01–1.41) (50).

Despite these cross-sectional study findings, a causal and pathogenic association between diabetes/prediabetes and e-cigarette use has not yet been established. To better investigate the effect of e-cigarette, use on the epidemiology of diabetes and prediabetes, longitudinal studies with enrolment of larger participant numbers is required to understand this relationship.

According to recent research, individuals who use e-cigarettes are at a more prominent risk of developing prediabetes (45). Specifically, the dual users have a higher prevalence of diabetes or prediabetes in comparison to individuals who smoke cigarettes exclusively (51). The rationale for this could be that the acute effect of nicotine has been demonstrated to cause a rise in glucose concentration in the blood (52). Additionally, in a study performed using data from the National Health and Nutrition Examination Survey, it was revealed that cotinine was correlated to more elevated levels of HbA1c in a group of individuals without diabetes (40). In addition to the effect of nicotine, there is evidence to indicate that the e-cigarette liquid itself may have harmful effects on glucose metabolism. Research conducted on rats demonstrated that exposure to e-cigarette liquid without nicotine resulted in hyperglycemia (3). A possible causal factor in the rise of sugar levels could be the presence of sugar, aldehydes, and other flavoring constituents within the e-liquid (44). The sole use of e-liquid can significantly impact the regulation of glucose levels in the body. The processes involved in changes to blood glucose levels after exposure to nicotine include glycogenolysis and gluconeogenesis.

### 4.4 E-cigarettes and metabolic syndrome

The use of e-cigarettes or using both, e-cigarettes and traditional cigarettes, among adults in the United States was associated with an increased risk of metabolic syndrome (MetS). Additionally, the prevalence of MetS was found to be higher in individuals who currently use e-cigarettes compared to those who used them in the past or never used them at all (53).

Similarly, a cross-sectional study among Palestinian university students found that cigarette and waterpipe smoking were linked to a higher risk of metabolic syndrome and elevated triglyceride levels, while black tea consumption was associated with lower risk. Although e-cigarette use was low, these findings highlight the broader impact of tobacco products on cardiometabolic health in young adults (54).

#### 4.4.1 Blood pressure

Studies investigating the direct effects of using e-cigarettes on blood pressure have reported short term rise in systolic and diastolic blood pressure briefly following exposure to e-cigarettes, particularly containing nicotine (55). These results suggest that there may be mechanisms, potentially involving other substances present in e-cigarette aerosols, that contribute to the elevation of blood pressure in addition to nicotine (56).

#### 4.4.2 LDL and HDL

Nicotine has significantly affected plasma triglyceride levels and HDL cholesterol levels (57). Effects of nicotine have been observed in animal studies and in humans who smoke cigarettes, indicating that nicotine leads to a rise in levels of triglyceride and a decline in the levels of HDL cholesterol (58). Furthermore, high levels of serum triglycerides are associated with a higher risk of premature atherosclerosis, hence the risk of cardiovascular effects. It is suggested that the inhalation of nicotine can induce lipolysis through the release of catecholamine, leading to abnormal serum lipids (51).

### 4.5 Cardiovascular effects

The regular use of e-cigarettes leads to a change in the balance of the autonomic nervous system toward a more significant prevalence of sympathetic activity, as well as heightened oxidative stress. These changes are known to be linked to an elevated risk of cardiovascular issues (59). While e-cigarettes retain lower amounts of carcinogens compared to traditional cigarettes (60), the substances present in e-cigarette aerosol can damage blood vessels and promote blood clot formation leading to risks of cardiovascular disease (61). The findings reveal that individuals who exclusively use e-cigarettes demonstrated elevated levels of fasting glucose, triglycerides, and reduced levels of HDL cholesterol. These results are indicative of an adverse cardiometabolic profile associated with e-cigarette usage (62). Therefore, the findings suggest an association between the use of e-cigarettes and the risk of cardiovascular effects.

A multicenter prospective randomized controlled trial protocol (DiaSmokeFree) was conducted to investigate whether transitioning from conventional combustible cigarettes to non-combustible nicotine alternatives, including electronic cigarettes and heated tobacco products, could affect cardiovascular and metabolic risk factors in individuals with T2DM in any way. However, the protocol does not provide clinical outcome data (63).

### 4.6 Public health implications and future directions

The findings synthesized in this review have important and under-recognized public-health implications. E-cigarettes are widely perceived as a lower-risk alternative to combustible tobacco, a narrative that has facilitated their rapid uptake among adolescents, young adults, and never-smokers. However, emerging evidence linking e-cigarette use to insulin resistance, dysglycemia, obesity, adverse lipid profiles, and components of metabolic syndrome suggests that potential harms extend beyond the respiratory and cardiovascular systems and into long-term metabolic health.

From a population perspective, even modest shifts in glycaemic control or cardiometabolic risk factors at the individual level can translate into substantial increases in the burden of type 2 diabetes, cardiovascular disease, and related complications when exposure is widespread. This concern is amplified by the fact that e-cigarette use is disproportionately prevalent among younger age groups, who may accumulate decades of exposure during critical developmental periods. Early metabolic disruption during adolescence and young adulthood is strongly associated with earlier onset of diabetes, accelerated atherosclerosis, and increased lifetime healthcare utilization, raising concerns that widespread vaping may contribute to a future rise in preventable metabolic disease.

The association between e-cigarette use and prediabetes observed in several large population-based datasets further highlights a gap in current screening and prevention strategies. At present, tobacco and vaping status is not routinely integrated into metabolic risk assessment or diabetes prevention programmes. Given the potential biological links between nicotine exposure, oxidative stress, GSK3β activation, and impaired insulin signaling, e-cigarette use may contribute to metabolic risk. Therefore, e-cigarette exposure should be considered when assessing metabolic risk, particularly among individuals with obesity, a family history of diabetes, or other cardiometabolic risk factors.

These findings also carry regulatory implications. Current regulatory frameworks largely focus on nicotine addiction, youth initiation, and pulmonary toxicity, with limited consideration of metabolic outcomes. The presence of flavoring agents, aldehydes, and solvents capable of inducing oxidative stress and metabolic dysregulation suggests that product standards based solely on nicotine content may be insufficient. Regulatory agencies may need to reassess permissible flavoring compounds, heating temperatures, and labeling requirements to reflect potential systemic metabolic risks, not just respiratory harm.

Public-health messaging should likewise evolve. While harm-reduction approaches may support e-cigarettes as a cessation aid for established smokers under controlled conditions, such messaging should be clearly distinguished from claims of metabolic or systemic safety. Communication strategies targeting adolescents and young adults should explicitly address emerging evidence linking vaping to insulin resistance, weight dysregulation, and cardiometabolic risk, countering the misconception that e-cigarettes are biologically inert outside the lungs.

The predominance of cross-sectional evidence underscores an urgent need for well-designed longitudinal studies to quantify long-term metabolic outcomes, dose–response relationships, and reversibility following cessation. Surveillance systems should incorporate biomarkers of glycaemic control, lipid metabolism, and insulin resistance alongside traditional cardiovascular endpoints. Without such data, public-health policy risks underestimating the true metabolic burden associated with e-cigarette use.

### 4.7 Limitations

This review has multiple limitations. Firstly, the evidence that is available is characterized by substantial heterogeneity in study designs, the populations, e-cigarette devices, as well as e-liquid formulations, concentrations of nicotine, exposure durations. This heterogeneity limits the direct comparisons across studies and thereby makes quantitative analysis challenging. Furthermore, most of the evidence is derived from observational and cross-sectional studies limiting the ability to identify whether e-cigarette exposure is directly associated with metabolic outcomes.

Additionally, self-reported e-cigarette use and the smoking history of individuals in some studies may also contribute to misclassification bias. Differences in device characteristics, e-liquid composition, and exposure conditions further limits direct comparison between preclinical and human studies. Moreover, the limited number of long-term longitudinal studies of e-cigarette restricts the long-term metabolic and cardiovascular consequences.

Therefore, the findings should be interpreted with consideration of the heterogeneity and limitations of the available evidence.

The study should incorporate comprehensive, up-to-date evidence, including studies published after the initial search. Findings should be interpreted cautiously given advancement of gene therapy and genome editing, as well as limited long-term clinical outcome data.

## 5 Conclusion

While e-cigarettes have been widely adopted as a perceived safer alternative to traditional smoking, emerging evidence suggests that their use may carry significant risks to metabolic health. The chemicals present in e-cigarette aerosols, particularly nicotine, have been shown to disrupt key metabolic processes, including glucose regulation and insulin signaling, which are critical for maintaining metabolic homeostasis. These disruptions could potentially increase the risk of metabolic disorders such as T2D and cardiovascular disease. As the popularity of e-cigarettes continues to rise, particularly among the younger demographic, it is imperative that public health policies and future research prioritize understanding the long-term metabolic effects of e-cigarette use. Comprehensive studies are needed to further elucidate these risks and to inform regulatory frameworks that can effectively mitigate potential health consequences. Addressing these gaps in knowledge is crucial for safeguarding public health and providing accurate information to consumers about the possible implications of e-cigarette use on metabolic health.
